# Identification of Influenza PA_N_ Endonuclease Inhibitors via 3D-QSAR Modeling and Docking-Based Virtual Screening

**DOI:** 10.3390/molecules26237129

**Published:** 2021-11-25

**Authors:** Chao Zhang, Junjie Xiang, Qian Xie, Jing Zhao, Hong Zhang, Erfang Huang, Pangchui Shaw, Xiaoping Liu, Chun Hu

**Affiliations:** 1 Key Laboratory of Structure-Based Drug Design & Discovery, Ministry of Education, Shenyang Pharmaceutical University, Shenyang 110016, China; zhangchaoylh@126.com (C.Z.); xiangjunjie97@163.com (J.X.); xqqx1996@163.com (Q.X.); zhaojing0507i@163.com (J.Z.); huang222fang@163.com (E.H.); lxp19730107@163.com (X.L.); 2 School of Life Science and Biopharmaceutics, Shenyang Pharmaceutical University, Shenyang 110016, China; 3 School of Life Sciences, The Chinese University of Hong Kong, Shatin, Hong Kong, China; pcshaw@cuhk.edu.hk

**Keywords:** 3D-QSAR, pharmacophore model, PA_N_ endonuclease, PA_N_ endonuclease inhibitors, raltegravir

## Abstract

Structural and biochemical studies elucidate that PA_N_ may contribute to the host protein shutdown observed during influenza A infection. Thus, inhibition of the endonuclease activity of viral RdRP is an attractive approach for novel antiviral therapy. In order to envisage structurally diverse novel compounds with better efficacy as PA_N_ endonuclease inhibitors, a ligand-based-pharmacophore model was developed using 3D-QSAR pharmacophore generation (HypoGen algorithm) methodology in Discovery Studio. As the training set, 25 compounds were taken to generate a significant pharmacophore model. The selected pharmacophore Hypo1 was further validated by 12 compounds in the test set and was used as a query model for further screening of 1916 compounds containing 71 HIV-1 integrase inhibitors, 37 antibacterial inhibitors, 131 antiviral inhibitors and other 1677 approved drugs by the FDA. Then, six compounds (**Hit01**–**Hit06**) with estimated activity values less than 10 μM were subjected to ADMET study and toxicity assessment. Only one potential inhibitory ‘hit’ molecule (**Hit01**, raltegravir’s derivative) was further scrutinized by molecular docking analysis on the active site of PA_N_ endonuclease (PDB ID: 6E6W). **Hit01** was utilized for designing novel potential PA_N_ endonuclease inhibitors through lead optimization, and then compounds were screened by pharmacophore Hypo1 and docking studies. Six raltegravir’s derivatives with significant estimated activity values and docking scores were obtained. Further, these results certainly do not confirm or indicate the seven compounds (**Hit01**, **Hit07**, **Hit08**, **Hit09**, **Hit10**, **Hit11** and **Hit12**) have antiviral activity, and extensive wet-laboratory experimentation is needed to transmute these compounds into clinical drugs.

## 1. Introduction

Influenza is an infectious disease caused by the influenza virus. It usually affects the upper respiratory tract and lungs. Seasonal and pandemic influenza viruses can be transmitted from animals to humans, which makes them particularly dangerous and leads to global health problems [1,2,3]. Though vaccines as a prophylactic have been recommended to reduce infection rate and epidemic possibility, it is not effective for everyone [4]. This may be due to the designing approaches of vaccines (such as live-attenuated or inactivated influenza vaccines) or the continual antigenic drift variation of influenza viruses. Moreover, it is not possible to generate prophylactic options against potential pandemic virus strains [5]. Three classes of clinical agents approved by the U.S. Food and Drug Administration (FDA), including agents that targeted the matrix 2 (M2) ion-channel, neuraminidase (NA), and the cap-snatching endonuclease activity of the polymerase acidic protein (PA), have been developed for the prophylaxis and treatment of influenza infection. However, the resistance to the M2 ion-channel inhibiting drugs makes amantadine and rimantadine invalidated their clinical utility [6,7]. It was also observed resistance to NA inhibitors (oseltamivir) in some seasonal influenza A strains [8,9]. Inhibitors of the cap-snatching endonuclease activity of PA have been made intensive research as a new series of anti-influenza inhibitors. Baloxavir marboxil is the first drug approved to target the cap-snatching endonuclease with a significant decrease in viral fitness [10,11]. Therefore, new drugs are essential for the treatment of drug-resistant and future pandemic flu strains.

Influenza A consisted of eight negative-stranded RNA genomic segments. The viral RNA-dependent RNA polymerase (RdRP) proteins were encoded by the three largest genomic RNA segments, the polymerase acidic protein (PA), polymerase basic protein 1 (PB1) and polymerase basic protein 2 (PB2) subunits. The influenza RdRP is essential for viral transcription and replication. It is also highly conserved among all influenza strains and subtypes. For the PA subunit, it has three main functions, which include endonuclease activity, involving in viral RNA (vRNA)/complementary RNA (cRNA) promoter binding and interacting with the PB1 subunit [12]. N-terminal fragments of PA (PA_N_, ≈25 kDa N-terminal domain; residues 1–197) and C-terminal fragments of PA (PA_C_, ≈55 kDa C-terminal domain; residues 239–716) are two domains of PA. The crystal structure of PA_C_ is solved in complexes with N-terminal fragments of PB1 (PB1_N_) [13]. The crystal structures of PA_N_ with both various ligands and unliganded have also been elucidated [14,15,16,17]. Biochemical and structural studies elucidated that PA_N_ might contribute to the host protein shutdown observed during influenza A infection [14,18,19]. Thus, inhibition of the endonuclease activity of influenza RdRP is an attractive target for novel antiviral therapy.

Computer aided drug design (CADD) is an important method of drug discovery, including virtual screening and pharmacophore design [20]. CADD can overcome certain difficulties from experiments in the laboratory via a virtual approach with a relatively low cost [21]. For example, Sourav et al. had built up a typical pharmacophore in the discovery of potential topoisomerase I inhibitors by ‘Common Features Pharmacophore’ techniques where the common features were only presented in the active compounds [22]. To generate the available pharmacophore, mostly active and moderately active compounds are considered as the training set molecules. In this manuscript, we collected 37 known PA_N_ endonuclease inhibitors [23,24,25,26] with diverse molecular structural patterns and created the training set and test set. We also constructed 10 pharmacophore models with activity prediction ability by utilizing the three-dimensional Quantitative Structure-Activity Relationship (3D-QSAR) Pharmacophore (HypoGen algorithm) technique. Then we screened a chemical database containing 1916 compounds (71 human immunodeficiency virus type 1 (HIV-1) integrase inhibitors [27], 37 antibacterial inhibitor, 131 antiviral inhibitors and 1677 approved drugs by FDA) based on the best pharmacophore Hypo1. Among the 1916 small-molecule inhibitors, part of them could form a chelate with two divalent metal ions, such as the HIV-1 integrase inhibitors. We assumed that they could also interact with the metal-chelating active site of influenza PA_N_ endonuclease well. Subsequently, we docked 6 selected compounds (**Hit01**–**Hit06**) with RNA endonuclease protein. At last, **Hit01** (raltegravir’s derivative) with significant screened results was selected as the ‘hit’ compound to target PA_N_ endonuclease for molecular optimization and transformation to obtain 197 novel pyrimidinone candidate molecules. According to the screening results based on the pharmacophore model Hypo1, six compounds with better estimated activity values than **Hit01** were selected. Our study may provide profound theoretical guidance and practical significance for the design and experimental synthesis of influenza virus inhibitors in the near future.

## 2. Result and Discussion

### 2.1. Pharmacophore Model Generation

**Figure 1:**
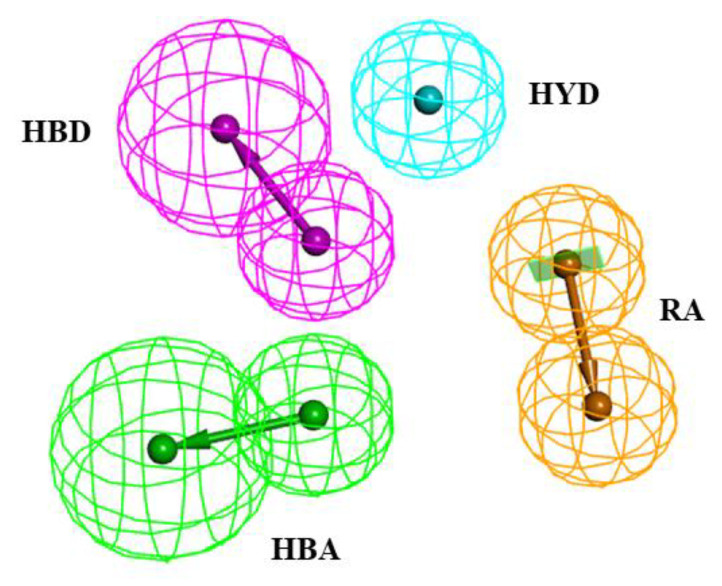
The best HypoGen Pharmacophore model (Hypo1). Green color represents HBA, purple color represents HBD, blue color represents HYD, brown color represents RA.

Chemically diverse 25 training set compounds containing active and moderately active compounds with corresponding IC_50_ values ranging from 0.011 μM to 37 μM, were selected to generate a pharmacophore model (Table 1) by Hypogen algorithm 3D-QSAR Pharmacophore protocol. The features of training set compounds, such as HBA, HBD, RA, HYD, PI and NI, were well distributed on the selected molecules (Figure 1). The significant statistical parameters such as cost, correlation coefficient, and RMSD of generated pharmacophore have been enlisted in Table 1. 10 pharmacophore models containing HBA, HBD, HYD and RA features were generated. The total cost of the generated pharmacophore models ranged from 130.623 to 172.486, with a null cost of 403.577 and a fixed cost of 71.464 bits. The difference between the null cost and total cost was used to describe the cost difference. The best hypothesis usually had the highest cost difference, a good correlation coefficient, the least RMSD, and a significant total cost value. Thus, the best pharmacophore Hypo1 enlisted in Table 1 was characterized by the lowest total cost value (130.623), the highest cost difference (272.95), the lowest RMSD (2.16926), and the best correlation coefficient (0.910618). The low RMSD and large correlation coefficient signified that Hypo1 had a better ability to predict the experimental activity of training set compounds.

**Figure 2:**
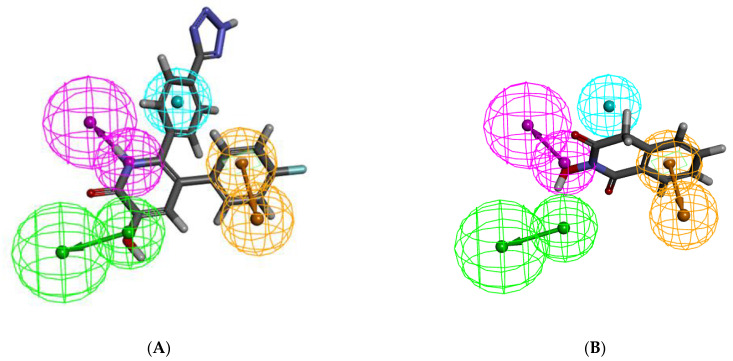
Pharmacophore mapping of the most active and less active compounds in the training set. (**A**) Hypo1 mapped on to the most active compound **T25**; (**B**) Hypo1 mapped on to the least active compound **T05**.

Furthermore, all the molecules selected from previously published research articles were divided into four groups of magnitude according to their experimental activity value (IC_50_) which were categorized in most active (≤0.1 μM, ++++), active (0.1 to 1.0 μM, +++), moderately active (1.0 to 10.0 μM, ++) and inactive (>10.0 μM, +). The experimental and estimated activity values of the training set compounds based on pharmacophore Hypo1 were shown in Table 2. In the training set, compound **T25** (Figure 2A) was estimated as the most active molecules as they were nicely mapped with all the essential features of the pharmacophore, whereas compound **T05** (Figure 2B) was considered as least active molecules due to the essential features were not mapped with Hypo1.

### 2.2. Validation of the Pharmacophore Models

The best pharmacophore model (Hypo1) was then validated by three distinct methods: (a) cost analysis, (b) Fischer’s randomization test and (c) test set analysis.

#### 2.2.1. Cost Analysis

The cost values of the total cost, fixed cost, and null cost were produced by the HypoGen algorithm in Discovery Studio and were utilized to analyze the ability to predict the experimental activity of training set compounds. If the cost difference was between 40 and 60 bits, the predicted correlation probability was assumed to be 75–90%. If the difference was greater than 60 bits, the predicted correlation probability was assumed to be more than 90%. The highest cost difference value of Hypo1 suggested that it could predict the experimental IC_50_ values of training set compounds with >90% statistical significance. The fixed cost displayed a model that fit all data perfectly. However, the null cost presumed that there was no relationship between the data and that the experimental activities were normally distributed around their average value. Thus, the significance of Hypo1 also depended on the total cost, fixed cost and null cost. In this study, the best hypothesis Hypo1 showed the fixed, total and null cost scores to be 71.464, 130.623 and 403.577, respectively.

#### 2.2.2. Fischer’s Randomization Test

**Figure 3:**
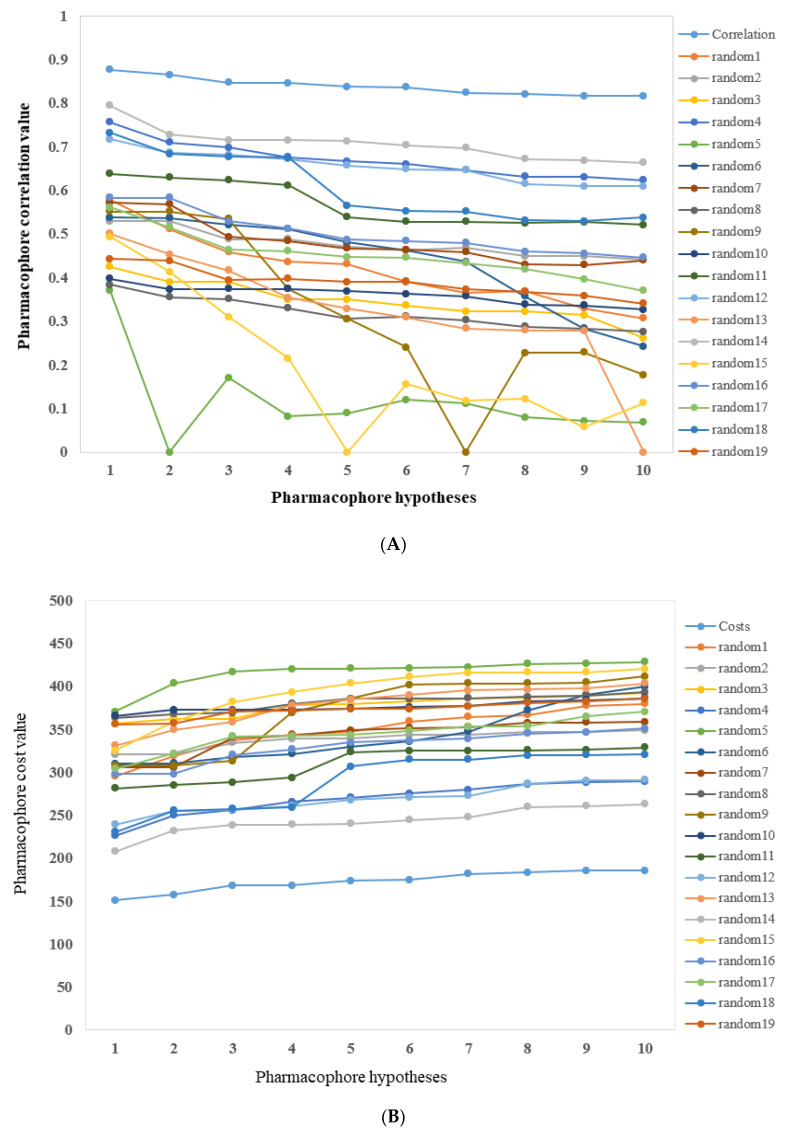
(**A**,**B**): The difference in correlation and total cost values of hypotheses between a Hypo1 spreadsheet and 19 random spreadsheets.

The experimental activity values of training set compounds were scrambled randomly. With a 95% confidence level, these values were used in pharmacophore generation and put forth 19 random spreadsheets. Then we compared these results with the originally generated pharmacophore (Hypo1). Figure 3 revealed the differences of correlations (Figure 3A) and costs values (Figure 3B) between the HypoGen and Fischer’s randomizations. None of the randomly generated pharmacophores obtained a better statistical value than Hypo1.

#### 2.2.3. Test Set Analysis

**Figure 4:**
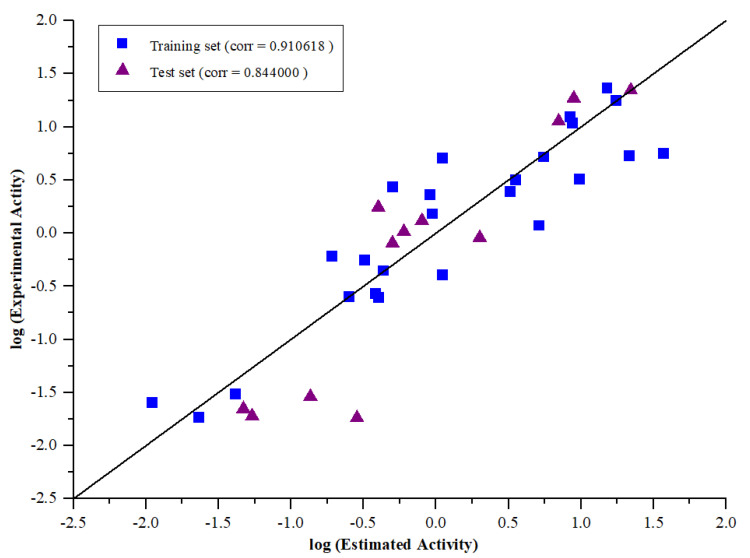
Correlation graph between experimental and estimated activity values in logarithmic scale for training and test set compounds based on Hypo1.

**Figure 5:**
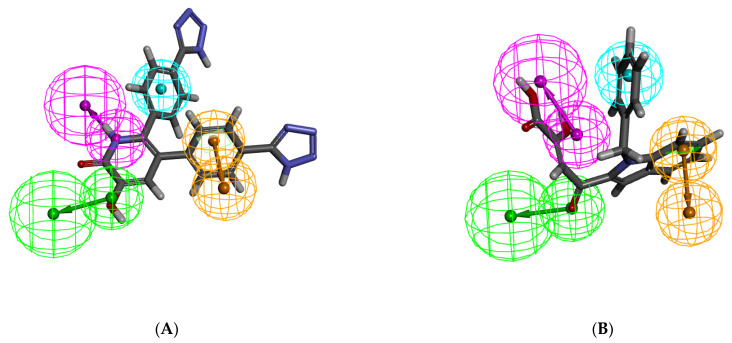
Pharmacophore mapping of the most active, less active compounds in the test set. (**A**) Hypo1 mapped on to the most active compound **T32**; (**B**) Hypo1 mapped on to the least active compound **T29**.

The reliability of the selected pharmacophore model was determined by its ability to predict the biological activities of test set compounds. 12 chemically diverse compounds were selected as test set, and their corresponding IC_50_ values ranged from 0.047 μM to 22 μM. To test the predictability ability of the pharmacophore model, we used the protocol in Discovery Studio to map the test set molecules. The estimated activity values were calculated for individual test set compounds and enlisted in Table 3. The correlation between experimental and estimated activity values was analyzed by simple regression. The strongest correlation coefficient between experimental and estimated PA_N_ endonuclease inhibitory activity values for the training set (r^2^ = 0.910618) and the test set (r^2^ = 0.844000) were shown in Figure 4. It was noticeable that the test set of 12 compounds was mapped properly with the generated pharmacophore. Among the 12 compounds, the best active compound **T32** was greatly mapped on the four essential features (Figure 5). The least active molecule **T29** did not map with the essential HBD feature, which signified the robustness of the pharmacophore model (Figure 5).

The employed three validation strategies exhibited that the Hypo1 model could be chosen as a significant pharmacophore model for further screening chemical databases with diverse structural entities.

### 2.3. Database Screening

Chemically novel and potential lead compounds could be possibly identified from the database containing 1916 compounds (71 HIV-1 integrase inhibitors [27], 37 antibacterial inhibitors, 131 antiviral inhibitors and 1677 other approved drugs by FDA) by virtual screening based on the generated pharmacophore [10]. Pharmacophore-based virtual screening was used to initiate the identification of novel scaffolds as PA_N_ endonuclease inhibitors and the validated pharmacophore Hypo1 was utilized as a 3D query for screening the database [18]. The diverse chemical databases were selected with the BEST search option to identify the prospective lead molecules. The potential lead compounds should fit with all the possible features of the validated pharmacophore Hypo1. Therefore, we obtained 16 molecules mapped on all features present in the Hypo1 model. We assumed that the most active compounds were estimated by activity value less than 10 μM. Subsequently, six compounds (**Hit01**–**Hit06**) were obtained with estimated activity of less than 10 μM (Supplementary Materials Table S1). Then we further evaluated them through ADMET and toxicity prediction.

### 2.4. ADMET and Toxicity Prediction

It would be beneficial to solve the problem which could cause a lead compound loss in preclinical and clinical trials, such as poor pharmacokinetic profile and toxic complications. The application of in-silico methodology for the prediction of the possible pharmacokinetic parameters and toxicity of the hit compounds would be advisable from an economic point of view. Then, the six compounds obtained after virtual screening were subjected to ADMET and various toxicity modules.

Compounds (**Hit01**–**Hit06**) were assessed by ADMET studies in Discovery Studio and the specific results were enlisted in Table 4. The solubility of these six compounds was accessible according to the results of ADME Solubility Level. The abilities to cross the blood–brain barrier (BBB) were medium or low, while **Hit01**, **Hit05** and **Hit06** were undefined. In addition, all of these compounds were not CYP2D6 inhibitors as the prediction results. Furthermore, these six compounds were easily absorbed and showed great plasma protein binding ability. Among the six compounds, **Hit02**, **Hit04** and **Hit05** were likely to be highly bound to carrier proteins in the blood.

The toxicity results of compounds (**Hit01**–**Hit06**) were enlisted in Table 5. NTP carcinogenicity prediction had been carried out on both female and male rats, and no compounds were found to be carcinogenic in nature on both male and female rats. Toxicity risk assessment results showed that compounds **Hit02**, **Hit03**, **Hit04** and **Hit05** might have carcinogenic properties against the male mouse while **Hit01** and **Hit06** are non-carcinogenic in nature. Non-carcinogen was found on the female mouse. Furthermore, the Ames mutagenicity and skin irritation tests had been performed against all the six potential hits and none of these potential hits showed any mutagenicity or skin irritation. Except for **Hit02** and **Hit06**, all the hit compounds did not exhibit developmental reproductive toxicity. Rat oral maximum lethal dose was also calculated for individual hit compounds and was enlisted in Table 5.

Based on the ADME and toxicity profiling, compound **Hit01** was regarded as the potential hit compound and was retained for docking study.

### 2.5. Molecular Docking Study

The molecular docking calculations were performed using Glide program in 2015 Schrödinger software package. The influenza A virus RNA polymerase complex (PDB ID: 6E6W) with 3-hydroxy-6-(2-methyl-4-(1*H*-tetrazol-5-yl)phenyl)-4-oxo-1,4-dihydropyridine-2-carboxylic acid (**T38**) [28] was chosen as the target protein for molecular docking. It was obtained from RCSB Protein Date Bank (RCSB PDB, http://www.rcsb.org (accessed on 7 November 2021).) and was prepared by Protein preparation wizard. The default values of the docking parameters were accepted. The active site was defined based on the co-crystal structure of **T38** bound to PA_N_ endonuclease complex. The docking results in our study were visualized using the PyMOL Molecular Graphics System (version 1.3).

Some important components, such as the binding modes, molecular interactions with the active site, binding energy and docking scores, were considered as the criterion in selecting the best poses of the docked compounds. In this study, docking compounds were ranked according to their docking scores, hydrogen bond interactions, and estimated activity. **T38** was chosen as the control compound to analyze the docking results and docked on the same active site of the PA_N_ endonuclease protein (PDB ID: 6E6W).

**Figure 6:**
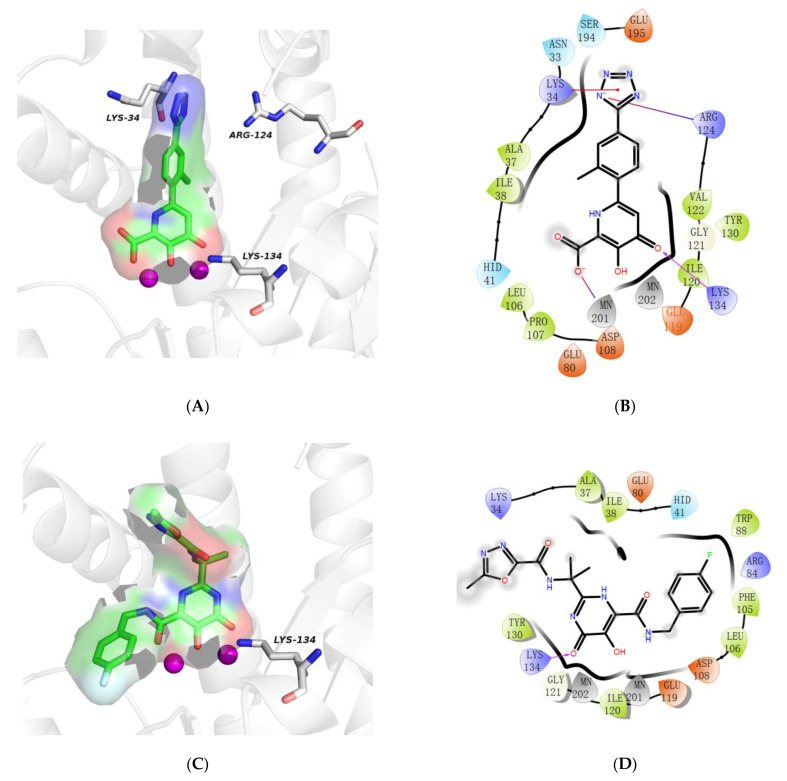
The binding interaction of **T38** and **Hit01** with PA_N_ endonuclease protein (PDB ID 6E6W). (**A**) The 3D docking pose of **T38** with 6E6W; (**B**) The 2D docking pose of **T38** with 6E6W; (**C**) The 3D docking pose of **Hit01** with 6E6W; (**D**) The 2D docking pose of **Hit01** with 6E6W.

The specific docking interactions of the two compounds (**T38** and **Hit01**) with 6E6W were enlisted in Table 6 and Figure 6. The filtered molecule bonded in the binding sites of PA_N_ endonuclease protein (PDB ID 6E6W) with docking scores of −6.622 kcal/mol. However, the docking score of **T38** with 6E6W was −8.227 kcal/mol. Previous studies demonstrated that the metal-chelating active site of PA_N_ was a negatively charged pocket, which consisted of a histidine (His41), a conserved lysine (Lys134), and a cluster of three acidic residues (Glu80, Asp108, and Glu119), and it could bind two divalent metal ions (Mn^2+^) [14,15,29,30,31,32,33,34]. It was noticeable that compounds **T38** and **Hit01** were located in the same pocket and generated hydrogen bonding with the same key amino acids Lys134 (1.9 Å and 2.8 Å). The N atom on tetrazole and oxygen atom on hydroxyl of carboxylic acid generated salt bridge with amino acids Arg124 and Mn^2+^, respectively. In addition, tetrazole ring generated Pi-cation interaction with Lys34. The analysis of the docking results and binding pattern implied that compound **Hit01** formed the same H-bond interaction with 6E6W compared with **T38**. The divalent metal ions (Mn^2+^ cations) present at the catalytic site were critical for the endonuclease activity. Interestingly, the interactions between divalent metal ions and two compounds (**T38** and **Hit01**) were similar to the interaction between diketo acid derivatives and divalent metal ions. The carboxylate of diketo acid (DKA) moiety chelates the first Mn^2+^ ion, while the second Mn^2+^ ion of PA_N_ interacts with the oxygen atom of its carboxylate moiety and the α-hydroxyl group, or the two Mn^2+^ ions simply form a chelate with the β-diketone group. Functional groups that chelate the Mn^2+^ ion of PA_N_ were crucial for inhibitors targeting the metal-chelating active site of PA_N_. This implied that pyrimidinone was an important moiety for interaction with divalent metal ions.

As the pyrimidinone derivative, raltegravir was initially approved by FDA in late 2007 as the first available agent targeted HIV integrase [35]. We considered **Hit01** (raltegravir’s derivative) as the hit compound and structured a compound database to detect more effective PA_N_ endonuclease inhibitors.

### 2.6. New Designed Compounds

**Figure 7:**
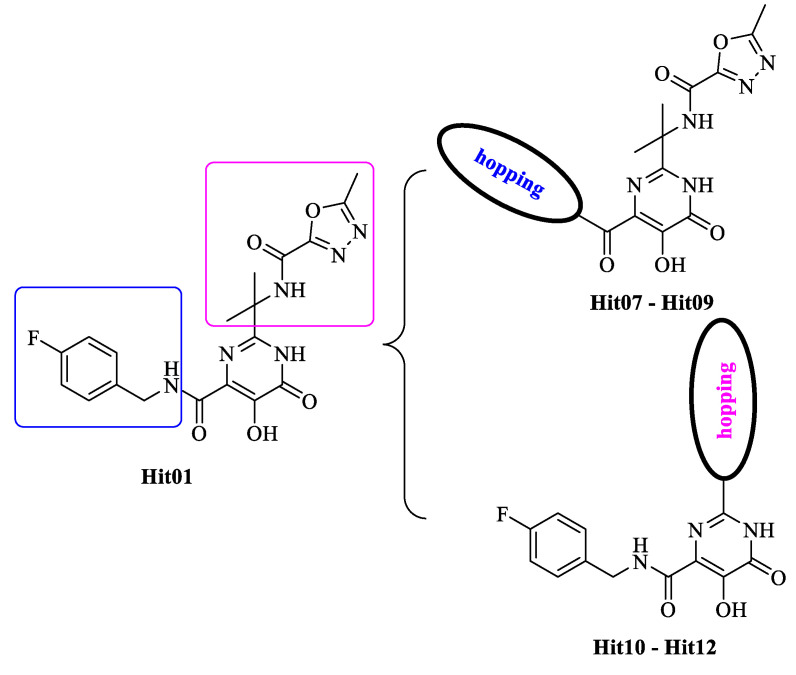
The pathway to elicit compounds (**Hit07**–**Hit12**) from template molecule (**Hit01**) using side-chain hopping.

We selected **Hit01** for Lead optimization, and 197 molecules were produced as derivatives. Subsequently, these 197 molecules were docked to the influenza PA_N_ endonuclease protein again to calculate the docking scores, estimate activity values, and fit values. The pathway to elicit compounds (**Hit07**–**Hit12**) from template molecule (**Hit01**) using side-chain hopping were shown in Figure 7. The top six PA_N_ inhibitors (**Hit07**–**Hit09** and **Hit10**–**Hit12**) were presented in Table 7 based on the molecular docking results and the predictable activity results.

The docking scores of selected candidate compounds (**Hit07**–**Hit09** and **Hit10**–**Hit12**) were lower than **Hit01**, except **Hit07** and **Hit11**. Furthermore, the estimated activity values of all compounds were more declined than **Hit01** (estimated activity value was 1.16424 μM) based on the prediction results. Table 7 enlisted the structures of the six raltegravir’s derivatives selected after Lead optimization and molecular docking. The functional groups were circled in blue and pink after Lead optimization and the control compound remained unchanged. It was noticeable that the six potent compounds which had good results in estimated activity could fit the values and docking scores after Lead optimization. The specific pharmacophore mapping and docking poses of the six compounds were shown in Supplementary Materials Figures S1 and S2.

Hence, **Hit01** and the novel six compounds (**Hit07**–**Hit09** and **Hit10**–**Hit12**) could be further developed and forwarded to be effective drugs for the treatment of influenza (Figure 7).

## 3. Materials and Method

### 3.1. Compound Preparations

**Figure 8:**
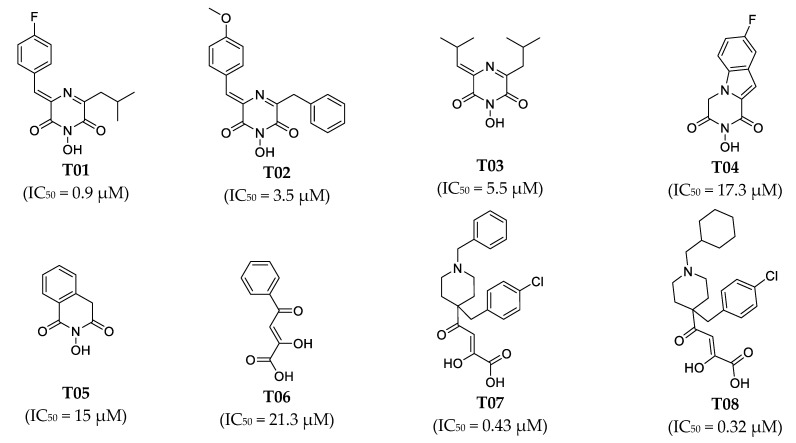

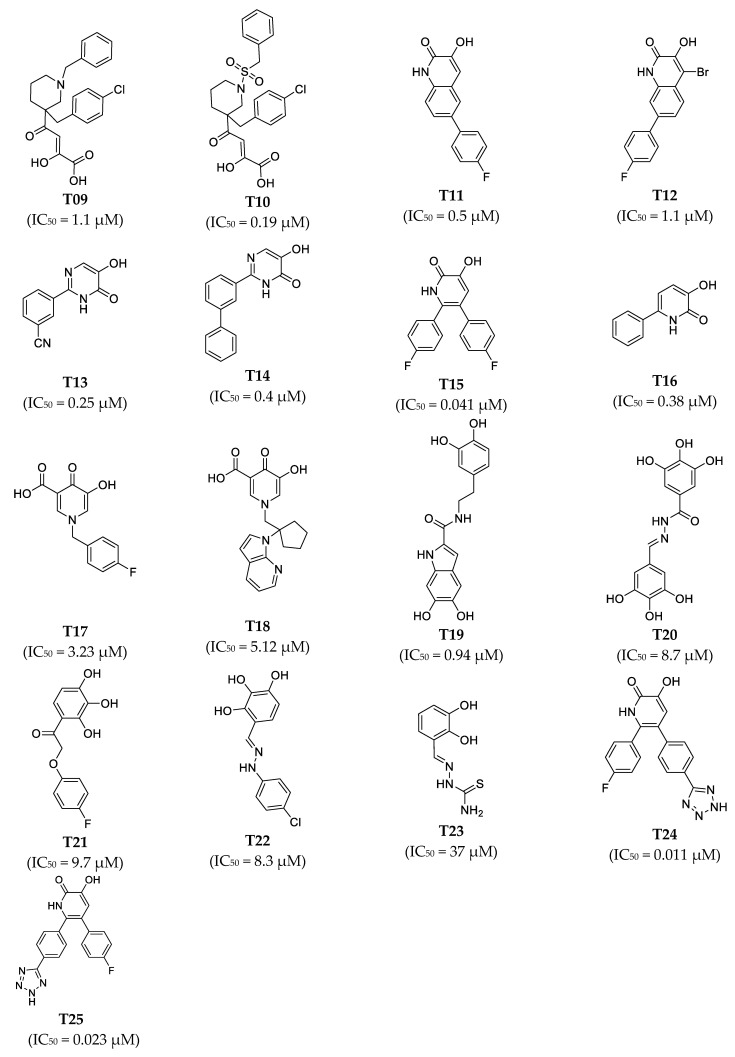
Chemical structures of PA_N_ endonuclease inhibitors in the training set together with their biological activity data (IC_50_ value, μM).

**Figure 9:**
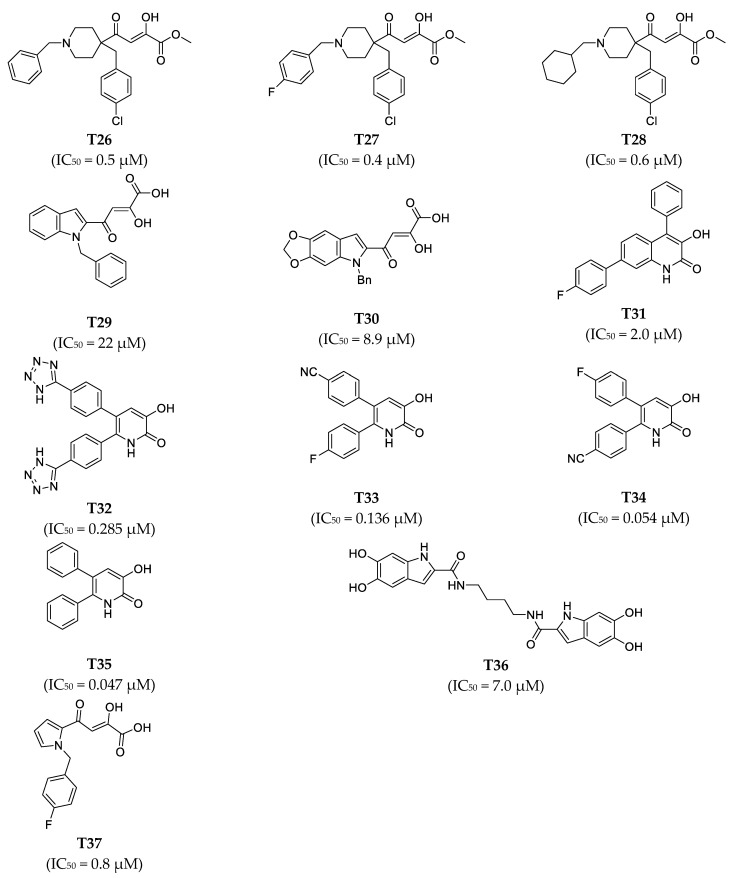
Chemical structures of PA_N_ endonuclease inhibitors in the test set together with their biological activity data (IC_50_ value, μM).

The two-dimensional (2D) structures of the 37 known PA_N_ endonuclease inhibitors were drawn with BIOVIA Draw 2016 [23,24,25,26]. Then, the 2D structures were converted into their corresponding 3D form using Discovery Studio [36]. We used the Generate Training and Test Data algorithm in Discovery Studio to split 37 objects into a training set and test set. The split method was set as random. Subsequently, 37 known PA_N_ endonuclease inhibitors with diverse molecular structural patterns were input into the “Input ligands” item. The training set percentage was set as 70 [37,38]. Subsequently, we obtained 25 training set compounds and 12 test set compounds. The structures of these training set compounds and test set compounds were given in Figure 8 and Figure 9, respectively. The training set was used to generate the pharmacophore model and the test set was used to evaluate the predictive ability of the generated pharmacophore model. The activity of training set (0.011 μM to 37 μM) and test set (0.047 μM to 22 μM) spanned over 5 orders.

We used Smart Minimizer algorithm for further minimization of each compound based on CHARMM force field method. In addition, each compound formed up to 255 different conformations in order to generate pharmacophore hypothesis or predict the activity of the newly found compounds [39,40,41].

### 3.2. Generation of Pharmacophore Models

The pharmacophore models which can be generated using 3D-QSAR Pharmacophore Generation protocol are correlated with the specific chemical features that are necessary for the biological activity of the molecules. We used the Feature Mapping protocol in Discovery Studio to seek the different chemical features presented on the training set molecules. These features, including hydrogen bond acceptor (HBA), hydrogen bond donor (HBD), hydrophobic (HYD), positive ionizable (PI), ring aromatic (RA) and negative ionizable (NI), were selected for the 3D-QSAR Pharmacophore Generation protocol. FAST algorithm method was applied to generate acceptable conformations for each compound with 10 kcal/mol as the energy threshold, and maximum generated conformations were set to 255. The uncertainty values of the training set and test set were set to 1.5 and the IC_50_ values of individual training set compounds were selected as an active property and the energy threshold was maintained at 20 kcal/mol during the pharmacophore generation. The minimum interfeature distance was changed from 2.97 to 2.0 and the maximum excluded volume was set to zero [2,42].

The pharmacophore models were generated according to significant statistical parameters such as total cost value, cost difference, error, root-mean-square deviation (RMSD), correlation coefficient (r^2^) and pharmacophore features.

### 3.3. Pharmacophore Validation

Cost analysis, Fischer’s randomization test and test set analysis are the three methods to validate the pharmacophore models.

There are three kinds of costs reported in Hypogen, including fixed cost, null cost and total cost. ∆Cost (Null cost–Total cost) is considered as a key parameter for the quality of the pharmacophore model. It infers a significant correlation if the cost difference is more than 60 bits. The model should fall in a prediction range of 70–90% if their cost difference is in the range of 40–60 bits regions. If the cost difference is less than 40 bits, it will be problematic during predicting correlation probability [43].

Fischer’s randomization technique acts as a fundamental role in making a correlation between the structural and biological activity in training set compounds. The validation of the selected pharmacophore hypothesis in this randomization technique produced 19 random spreadsheets in 95% confidence levels by shuffling the activity values of the training set compounds [44,45]. The processes of building and minimizing all test set compounds are similar to that of all training set molecules. The test set with 12 molecules was selected to validate the pharmacophore model. Ligand Pharmacophore Mapping protocol in Discovery Studio was utilized to overlap the validated pharmacophore with the active molecules [46].

### 3.4. Database Screening

Well validated pharmacophore Hypo1 was used as a 3D query for identifying potential leading molecules from a chemical database which containing 71 HIV-1 integrase inhibitors [27] as previously reported and additional 1845 drugs (37 antibacterial inhibitors, 131 antiviral inhibitors and 1677 other approved drugs by FDA) that obtained from ZINC database (ZINC, http://zinc.docking.org (accessed on 7 November 2021).). The fast search method was carried out to obtain the ‘hit’ compounds matching with the features in the best pharmacophore Hypo1. Then, the filtered molecules with estimated activity values <10.0 μM were implied for further screening of absorption, distribution, metabolism, excretion and toxicity (ADMET) properties.

### 3.5. Determination of In-Silico Pharmacokinetic Properties

Screened and selected compounds were subjected to analyze the ADMET properties and the drug-likeness. Nowadays, a popular method to predict ADMET properties is the in silico methodology, such as Discover Studio 2016 ADMET PREDICT [47]. Although there are certain limitations, it is a vital method to cut down the actual costs for in vitro analysis. In this study, we used Discover Studio 2016 ADMET PREDICT to analyze the ADMET properties and the drug-likeness. Then, the compounds with better ADME properties and lower toxicity were subjected to the following molecular docking study.

### 3.6. Molecular Docking

The Gild-XP (extra precision) was applied to perform virtual screening and study the interactions between the selected molecular candidates and the protein structure [48,49]. We selected the influenza PA_N_ endonuclease protein (PDB ID: 6E6W) based on the previous literature sources [22,23,24,25,26] and retrieved protein crystal structure (PDB ID: 6E6W) from the RCSB Protein Data Bank (RCSB PDB, http://www.rcsb.org/ (accessed on 7 November 2021)). Schrödinger’s protein preparation wizard was used to remove the cofactors, co-crystallized ligand and water molecules. We also used it to add missing residues and hydrogens, generate Het states and optimize the selected protein [39]. The prepared protein structure was further processed for grid generation [50]. The active site was decided by default parameters like centroid of inbound co-crystal ligand and looking at key residues of in-bound ligand. The box size was set to 20 Å. The selected ligands from the results of 3D-QSAR models were implied to the molecular docking by Gild-XP (extra precision). The docking results were ranked on the basis of the Gild score, and the top-ranked molecules were visually analyzed.

### 3.7. New Designed Compounds

In the protocol of Replace Fragment, **Hit01** was selected as the ‘hit’ compound, and we hop its side-chain by fusing the same fragments onto the molecules to improve the activity. Keeping the original molecular skeleton (Pyrimidinone), 4-fluorobenzyl and 1,3,4-oxadiazole-2-carboxamide were named as Group and Group 1 to imply side-chain hopping. The fragment library was set as the default parameter. The Generate fragment conformation and Parallel processing were applied. The designed compounds, which fit all the possible features of the validated pharmacophore Hypo1, were docked back to the active site of PA_N_ endonuclease again for evaluating binding poses and binding affinity.

## 4. Conclusions

This study provided the development of ligand-based pharmacophore model by 3D-QSAR Pharmacophore Generation protocol using Discovery Studio.

25 diverse compounds were considered as a training set for the development of the new pharmacophores model. We selected the best quantitative pharmacophore (Hypo1) from 10 other pharmacophores based on the highest cost difference (272.95), lowest total cost value (130.623) and best correlation coefficient (0.910618). The selected Hypo1 model consisted of four features (HBA, HBD, HYD and RA) and had been cross-validated by cost analysis, test set predictions, and Fischer’s randomization test. The test set utilized for evaluating the predictive ability of Hypo1 model consisted of 12 compounds. Then, we obtained the resulting correlation coefficient between the estimated activity and experimental activity for the 12 test set compounds, which was 0.844000. The Hypo1 model was used as a 3D query for the virtual screening of 1916 compounds (71 HIV-1 integrase inhibitors, 37 antibacterial inhibitors, 131 antiviral inhibitors and 1677 other approved drugs by FDA). The 6 compounds with estimated activity less than 10 μM were hit. The 6 compounds were subjected to further ADMET studies and were carried out toxicity assessment studies under TOPKAT program to obtain candidate compounds. Subsequently, **Hit01** was selected based on the filtration and retained for docking study, which exhibited better docking scores than the reported control compound (**T38**) according to the docking studies. Then we compared the docking scores and interaction with the active site residues of **Hit01** (−6.622 kcal/mol) with the standard compound (**T38**). Based on our findings, the hit compound (**Hit01**) was utilized for designing a future class of potential PA_N_ endonuclease inhibitors. **Hit01** was gone for lead optimization, and then 197 molecules were produced as derivatives. After lead optimization, 6 potent compounds obtained good results in estimate activity, fit values and docking scores. Therefore, we speculate that these 7 compounds (**Hit01**, **Hit07**, **Hit08**, **Hit09**, **Hit10**, **Hit11** and **Hit12**) will target PA_N_ endonuclease to exhibit good anti-influenza virus activity. Extensive wet-laboratory experimentation is needed to transmute these seven pyrimidinone derivatives (**Hit01**, **Hit07**, **Hit08**, **Hit09**, **Hit10**, **Hit11** and **Hit12**) into clinical drugs.

## Figures and Tables

**Table 1:** Statistical results of the top 10 pharmacophore hypotheses generated by HypoGenalgorithm.

Hypo. No.	Total Cost	Cost Difference	RMSD	Correlation	Max.Fit	Features
1	130.623	272.95	2.16926	0.910618	7.00748	HBA, HBD, HYD, RA
2	156.57	247.01	2.60686	0.867964	7.31818	HBA, HBA, HBD, HYD
3	157.184	246.39	2.5833	0.870548	5.4078	HBD, HBD, HYD, RA
4	160.073	243.50	2.62325	0.866204	5.25001	HBD, HBD, HYD, HYD
5	165.706	237.87	2.74058	0.852888	6.97954	HBA, HBA, HYD, RA
6	168.233	235.34	2.74498	0.852405	5.2546	HBD, HBD, HYD, HYD
7	168.516	235.06	2.77498	0.848851	6.47651	HBA, HBD, HBD, HYD
8	171.636	231.94	2.82585	0.842729	6.98225	HBA, HBD, HYD, RA
9	172.439	231.14	2.81172	0.84447	5.49327	HBA, HBD, HYD, RA
10	172.486	231.09	2.82443	0.842919	6.04898	HBA, HBA, HYD, RA

Null cost = 403.577, Fixed cost = 71.464, Best record in pass = 6, Configuration cost = 11.168. (RA: ring aromatic, HBA: hydrogen bond acceptor, HBD: hydrogen bond donor, HYD: hydrophobic).

**Table 2:** Experimental and Estimate activity of the training set compounds based on pharmacophore Hypo1.

Comp. No.	IC_50_ Value (μM)		Errors ^a^	Fit Value ^b^	Activity Scale ^c^	
	Experimental	Estimated			Experimental	Estimated
**T01**	0.9	2.4	2.6	4.26	+++	++
**T02**	3.5	3.2	−1.1	4.12	++	++
**T03**	5.5	5.3	−1	3.91	++	++
**T04**	17.3	18	1	3.38	+	+
**T05**	15	23	1.5	3.26	+	+
**T06**	21.3	5.4	−4	3.9	+	++
**T07**	0.43	0.44	1	4.99	+++	+++
**T08**	0.32	0.56	1.7	4.89	+++	+++
**T09**	1.1	0.41	−2.7	5.02	++	+++
**T10**	0.19	0.6	3.2	4.85	+++	+++
**T11**	0.5	2.7	5.5	4.19	+++	++
**T12**	1.1	5.1	4.7	3.92	++	++
**T13**	0.25	0.25	1	5.22	+++	+++
**T14**	0.4	0.25	−1.6	5.23	+++	+++
**T15**	0.041	0.031	−1.3	6.14	++++	++++
**T16**	0.38	0.27	−1.4	5.19	+++	+++
**T17**	3.23	2.5	−1.3	4.23	++	++
**T18**	5.12	1.2	−4.3	4.55	++	++
**T19**	0.94	1.5	1.6	4.44	+++	++
**T20**	8.7	11	1.2	3.6	++	+
**T21**	9.7	3.4	−2.9	4.1	++	++
**T22**	8.3	13	1.5	3.53	++	+
**T23**	37	5.7	−6.5	3.88	+	++
**T24**	0.011	0.026	2.3	6.22	++++	++++
**T25**	0.023	0.019	−1.2	6.35	++++	++++

^a^ Error factor calculated as the ratio of the measured activity to the estimated activity; positive value indicates that the estimated IC_50_ is higher than the experimental IC_50_; a negative value indicates that the estimated IC_50_ is lower than the experimental IC_50_ value. ^b^ Fit value indicates how well the features in the pharmacophore map with the chemical features present in the compound. ^c^ Activity scale: ++++, IC_50_ ≤ 0.1 μM (most active); +++, IC_50_ 0.1 to 1.0 μM (active); ++, IC_50_ 1.0 to 10.0 μM (moderately active); +, IC_50_ > 10.0 μM (inactive).

**Table 3:** Experimental and estimated activity of the test set compounds based on pharmacophore Hypo1.

Comp. No.	IC_50_ Value (μM)		Errors ^a^	Fit Value ^b^	Activity Scale ^c^	
	Experimental	Estimated			Experimental	Estimated
**T26**	0.5	0.81	1.6	4.72	+++	+++
**T27**	0.4	1.8	4.4	4.39	+++	++
**T28**	0.6	1.0	1.7	4.62	+++	++
**T29**	22	22	1.0	3.28	+	+
**T30**	8.9	19	2.1	3.36	++	+
**T31**	2.0	0.91	−2.2	4.67	++	+++
**T32**	0.285	0.018	−16	6.37	+++	++++
**T33**	0.136	0.029	−4.7	6.17	+++	++++
**T34**	0.054	0.019	−2.8	6.35	++++	++++
**T35**	0.047	0.022	−2.1	6.28	++++	++++
**T36**	7.0	11	1.6	3.58	++	+
**T37**	0.8	1.3	1.6	4.51	+++	++

^a^ Error factor calculated as the ratio of the measured activity to the estimated activity; positive value indicates that the estimated IC_50_ is higher than the experimental IC_50_; a negative value indicates that the estimated IC_50_ is lower than the experimental IC_50_ value. ^b^ Fit value indicates how well the features in the pharmacophore map with the chemical features present in the compound. ^c^ Activity scale: ++++, IC_50_ ≤ 0.1 μM (most active); +++, IC_50_ 0.1 to 1.0 μM (active); ++, IC_50_ 1.0 to 10.0 μM (moderately active); +, IC_50_ > 10.0 μM (inactive).

**Table 4:** ADMET descriptors of the selected candidates.

Comp. No.	ADME Solubility Level	ADME BBB Level	ADME Absorption Level	CYP2D6 Prediction	PPB Prediction
**Hit01**	3	4	3	false	false
**Hit02**	3	3	0	false	true
**Hit03**	2	3	0	false	false
**Hit04**	3	2	0	false	true
**Hit05**	2	4	1	false	true
**Hit06**	3	4	0	false	false

ADME_Solubility_Level: 0 (Extremely low); 1 (No, very low, but possible); 2 (Yes, low); 3 (Yes, good); 4 (Yes, optimal); 5 (No, too soluble); 6 (Warning: molecules with one or more unknown AlogP98 types). ADME_BBB_Level: 0 (Very high penetrant); 1 (High); 2 (Medium); 3 (Low); 4 (Undefined). ADME_Absorption_Level: 0 (Good absorption); 1 (Moderate absorption); 2 (Low absorption); 3 (Very low absorption). EXT_CYP2D6_Prediction: false: non-inhibitor; true: inhibitor. EXT_PPB_Prediction: plasma protein binding ability, false: ≥90%; true: ≤90%.

**Table 5:** Toxicity Predictions of the lead molecules by TOPKAT.

Comp. No.	Hit01	Hit02	Hit03	Hit04	Hit05	Hit06
NTP carcinogenicity male Rat	NC	NC	NC	NC	NC	NC
NTP carcinogenicity female Rat	NC	NC	NC	NC	NC	NC
NTP carcinogenicity Call (Male mouse)	NC	C	C	C	C	NC
NTP carcinogenicity Call (Female mouse)	NC	NC	NC	NC	NC	NC
Ames mutagenicity	NM	NM	NM	NM	NM	NM
Developmental Toxicity Potential (DTP)	NT	T	NT	NT	NT	T
Rat oral LD_50_ (in g/kg)	0.39282	0.183468	0.627103	0.216087	0.744138	2.43894
Skin irritation	None	None	None	None	None	None

C: Carcinogen; NC: Non-Carcinogen; NM: Non-Mutagen; NT: Non-Toxic; T: Toxic.

**Table 6:** Docking interactions of **T38** and **Hit01** with PA_N_ endonuclease protein (PDB ID 6E6W).

Comp. No.	H-Bond Interaction	H-Bond Distance (Å)	Docking Score (kcal/mol)
**T38**	Lys134	1.9	−8.227
**Hit01**	Lys134	2.8	−6.622

**Table 7:** Structures of selected candidate compounds (**Hit07**–**Hit09** and **Hit10**–**Hit12**) with Estimate activity, Fit value and docking scores.

Comp. No.	Structure	Estimate	Fit Value	Docking Scores (kcal/mol)
**Hit07**	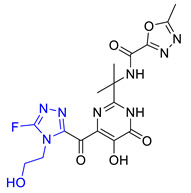	0.12478	5.53416	−5.791
**Hit08**	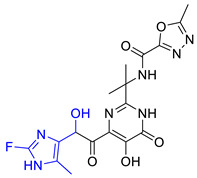	0.13246	5.50822	−10.803
**Hit09**	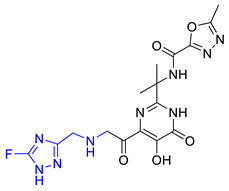	0.159602	5.42726	−8.626
**Hit10**	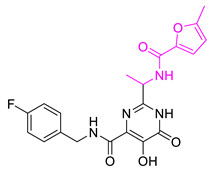	0.800934	4.7267	−8.944
**Hit11**	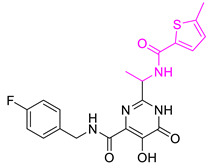	1.03327	4.61609	−7.131
**Hit12**	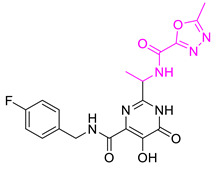	1.08169	4.5962	−9.938

## Data Availability

The data presented in this study are available on request from the corresponding authors.

## Supplementary Materials

The following are available online. The structures, estimated activity, specific pharmacophore mapping and docking poses of the hit compounds were supported in Table S1, Figures S1 and S2.

## Sample Availability

No chemical samples are available.
