# Supplementary material for: Identification of Influenza PAN Endonuclease Inhibitors via 3D-QSAR Modeling and Docking-Based Virtual Screening
Source: Molecules. 2021 Nov 25;26(23):7129. doi: 10.3390/molecules26237129 (PMC8659138; doi:10.3390/molecules26237129)
Supplement: Supplementary file 1 [file molecules-26-07129-s001.zip › molecules-1430463-supplementary.pdf]

molecules

Supplementary Materials

Identification of influenza PAN endonuclease inhibitors via 3D-QSAR Modeling and docking-based virtual screening

Chao Zhang [1], Jun-Jie Xiang [1], Qian Xie [1], Jing Zhao [1], Hong Zhang [2,*], Er-Fang Huang [1], Pang-Chui Shaw [3], Xiao-Ping Liu [1] and Chun Hu [1,*]

1 Key Laboratory of Structure-Based Drug Design & Discovery, Ministry of Education; Shenyang Pharmaceutical University, Shenyang 110016, China; zhangchaoylh@126.com (C. Zhang); xiangjunjie97@163.com (J. Xiang); xqqx1996@163.com (Q. Xie); zhaojing0507i@163.com (J. Zhao); huang222fang@163.com (E. Huang); lxp19730107@163.com (X. Liu); chunhu@syphu.edu.cn (C. Hu).
2 School of Life Science and Biopharmaceutics, Shenyang Pharmaceutical University, Shenyang 110016, China; song0688@sina.com (H. Zhang).
3 School of Life Sciences, The Chinese University of Hong Kong, Shatin, Hong Kong SAR, China; pcshaw@cuhk.edu.hk (P. Shaw).

* Correspondences: chunhu@syphu.edu.cn (C. Hu) & song0688@sina.com (H. Zhang); Tel.: +86-24-43520246 (C. Hu).

molecules MDPI

Table S1. Structures of selected candidate compounds (**Hit 1 – Hit 6**) with Estimate activity and Fit value.

Compound	Structure	Estimated IC_{50} value (μM)	Fit value
**Hit01**	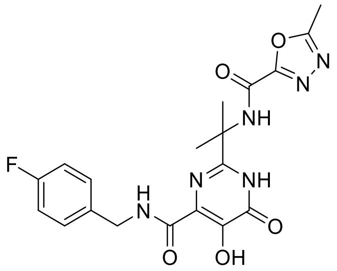	1.16424	4.56426
**Hit02**	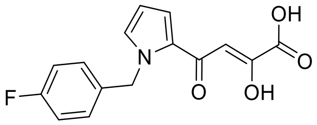	1.31451	4.51154
**Hit03**	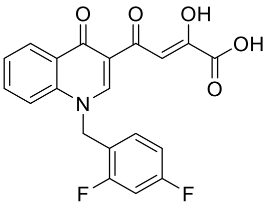	0.158572	5.43007
**Hit04**	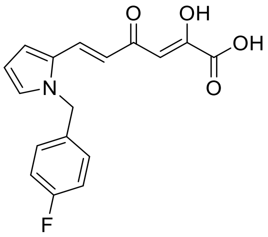	0.942881	4.65584
**Hit05**	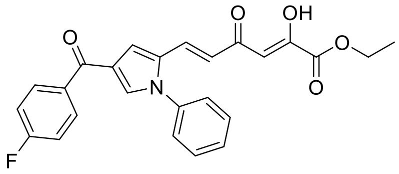	1.97529	4.33467
**Hit06**	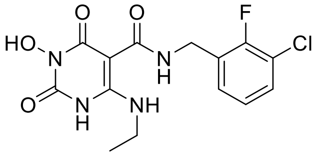	4.63103	3.96462

We carried out active prediction using the generated Hypo1 pharmacophoric model. It was obviously to see that the novel 6 compounds mapped well with all chemical features (Figure S1), including one hydrogen bond acceptor (HBA), one hydrogen bond

donnor (HBD), one hydrophobic (HYD) and one ring aromatic (RA). And the estimated activities were extremely lower than the lead compounds.

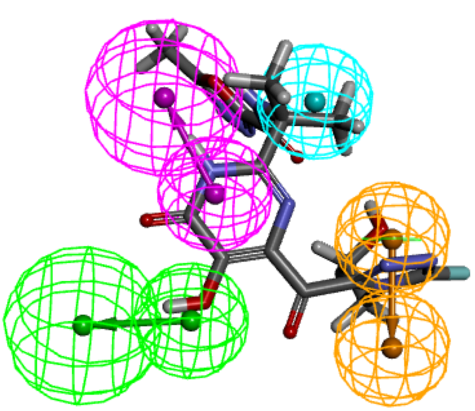

Hit07 – Hypo1

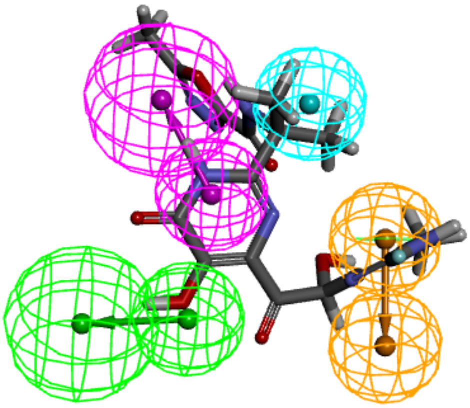

Hit08 – Hypo1

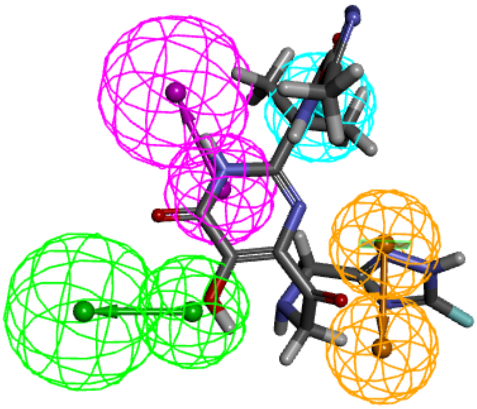

Hit09 – Hypo1

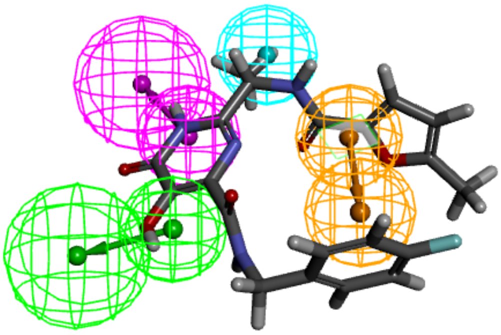

Hit10 – Hypo1

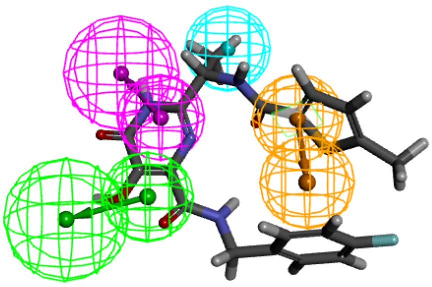

H

it11 – Hypo1

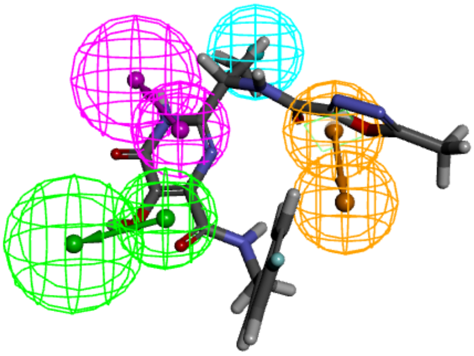

Hit12 – Hypo1

Figure S1. Pharmacophore mapping of the six compounds.

molecules MDPI

Then we selected typical compounds (**Hit08** and **Hit10**) to analyze the docking poses using Glide program in 2015 Schrödinger software package. The specific docking poses were showed in Figure S2.

Compound **Hit08** (N-{2-{4-[2-(2-fluoro-5-methyl-1H-imidazol-4-yl)-2-hydroxyacetyl]-5-hydroxy-6-oxo-1,6-dihydropyrimidin-2-yl}propan-2-yl}-5-methyl-1,3,4-oxadiazole-2-carboxamide) was found to interact with amino acids Lys34 and Lys134, which were all essential binding site residues. The carbonyl oxygen atom in pyrimidinone formed one hydrogen bond interactions with amino acids Lys 134. The 1,3,4-oxadiazole generated Pi-cation bond with amino acids Lys34. Furthermore, the Hydroxyl oxygen atom in pyrimidinone generated salt bridge with amino acids Lys134 (Fig. 10A). The first Mn^{2+} ion of PA_N interacts with the oxygen atom of its carboxylate moiety and the α-hydroxyl group while the carboxylate of pyrimidinone moiety chelates the second Mn^{2+} ion.

Compound **Hit10** (N-(4-fluorobenzyl)-5-hydroxy-2-[1-(5-methylfuran-2-carboxamido)ethyl]-6-oxo-1,6-dihydropyrimidine-4-carboxamide) was able to form one hydrogen bond interaction with amino acid Leu106. The furan ring generated Pi-cation bond with amino acids Lys34. Hydroxyl oxygen atom in pyrimidinone also generated salt bridge with amino acids Lys134. The carboxylate of pyrimidinone moiety chelates the first Mn^{2+} ion, while the second Mn^{2+} ion of PA_N interacts with the oxygen atom of its amide moiety and the α-hydroxyl group of pyrimidinone moiety.

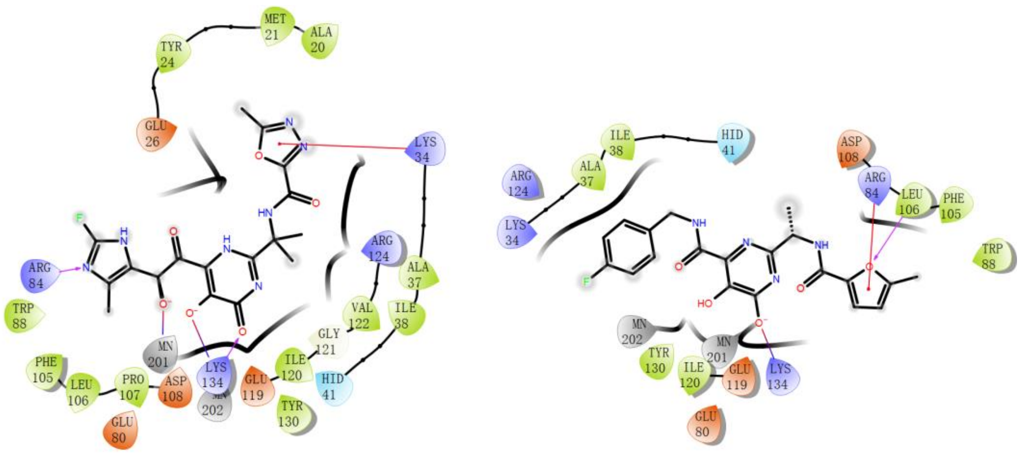

Hit08 – 6E6W **Hit10**– 6E6W

Figure S2. The binding interactions of **Hit08** and **Hit10** with PA_N endonuclease protein (PDB ID 6E6W)
